# Preliminary Study of the Potential Extracts from Selected Plants to Improve Surface Cleaning

**DOI:** 10.3390/plants7010017

**Published:** 2018-03-06

**Authors:** Ai Ting Vong, Hui Wen Chong, Vuanghao Lim

**Affiliations:** 1 Oncological and Radiological Sciences Cluster, Advanced Medical and Dental Institute, Universiti Sains Malaysia, Bertam, Kepala Batas 13200, Penang, Malaysia; aitingv@gmail.com; 2 Integrative Medicine Cluster, Advanced Medical and Dental Institute, Universiti Sains Malaysia, Bertam, Kepala Batas 13200, Penang, Malaysia; c_hwen91@hotmail.com

**Keywords:** antimicrobial, Cinnamomum iners, Citrus hystrix, Citrus aurantifolia, aqueous extract

## Abstract

Environment hygiene is important for preventing infection and promoting a healthier environment in which to live or work. The goal of this study was to examine the antimicrobial effects of *Citrus aurantifolia* (key lime) juice and aqueous extracts of *Cinnamomum iners* (cinnamon) bark and *Citrus hystrix* (kaffir lime) leaves on the kinetic growth of *Pseudomonas aeruginosa* and methicillin resistance *Staphylococcus aureus* (*MRSA*). Antimicrobial activity was quantitatively evaluated using spectrophotometry and viable cell counts versus bacterial growth time. The fomite surface samples that were used in the second experiment were chosen randomly from the laboratories. They were assessed both before and after intervention using a mixture of commercial disinfectant detergent and lime juice. In the kinetic growth study, the lime juice effectively eliminated *P. aeruginosa* and *MRSA*. The cinnamon bark extract was more effective at inhibiting *P. aeruginosa* than *MRSA.* The kaffir lime leaf extract demonstrated bacteriostatic activity for the first 60 min, which then weakened after 90 min for both bacteria. The lime juice extract and commercial disinfectant mixture effectively disinfected the fomites. Further studies of the use of key lime juice as a disinfectant in the hospital environment should be conducted, as *C. aurantifolia* exhibits antibacterial activities against endemic microbes.

## 1. Introduction

Indoor cleanliness should be maintained to a standard level to preclude surface degradation and to control the potential risk of infection and dust exposure. Environment surfaces, such as floors and tiles, are often covered by a layer of dust consisting of minerals, metals, fibres, particles, organic compounds, polycyclic aromatic compounds, and biological entities, including bacteria, pollen, and animal allergens. Disinfection via chemical or thermal action is essential in the cleaning process that provides broad microbiological sterilisation. Chemical disinfection is often the preferred choice for disinfecting and maintaining fomite surfaces [1]. The degree of bactericidal action against microorganisms depends on their sensitivity to the disinfectant chemical. A high level disinfectant helps eliminate microorganisms, excluding large quantities of spores, whereas low level disinfection is inefficient for mycobacteria or spore clearance [2].

A variety of cleaning agents have been formulated to aid in dust and dirt removal, disinfection, and surface maintenance [3]. The cleaning process involves chemical reactions, whereby deposits of minerals or inorganic salts are dissolved via acid-base reactions or complex formation or formation of micelles to remove dirt or lipid components [3]. The process also can reduce the microorganism load on the surface. The three types of cleaning are use of detergents, disinfectants, or the combination of the two. Detergents are effective at removing organic material and suspended grease or oil, whereas disinfectants can reduce or eliminate microorganisms. Detergent-disinfectants are capable of both functions [2], and the combination helps decrease the microorganism load significantly and quickly.

A wide spectrum of exposure to chemical agents can cause severe health problem, such as allergies, eczema, and asthma. Allergic response can be activated via skin contact or inhalation even if only small amounts of allergen are present in a disinfectant [3,4]. Exposure to chemical agents depends greatly on the quantity of residues, degree of off-gassing, and the amount of dissolved surface elements [3]. As disinfectant-detergents are formulated in concentrated solution and often in aerosol form, users should wear gloves when handling. Due to the potential health risks posed by chemical agents, alternative surface cleaning agents are needed. The goal of this study was to evaluate the potential for *Citrus aurantifolia* (key lime) juice and aqueous extracts of *Cinnamomum iners* (cinnamon) bark and *Citrus hystrix* (kaffir lime) leaves to eliminate bacteria effectively from surfaces.

## 2. Results

### 2.1. Preliminary Screening of Plants Extracts

**Figure 1:**
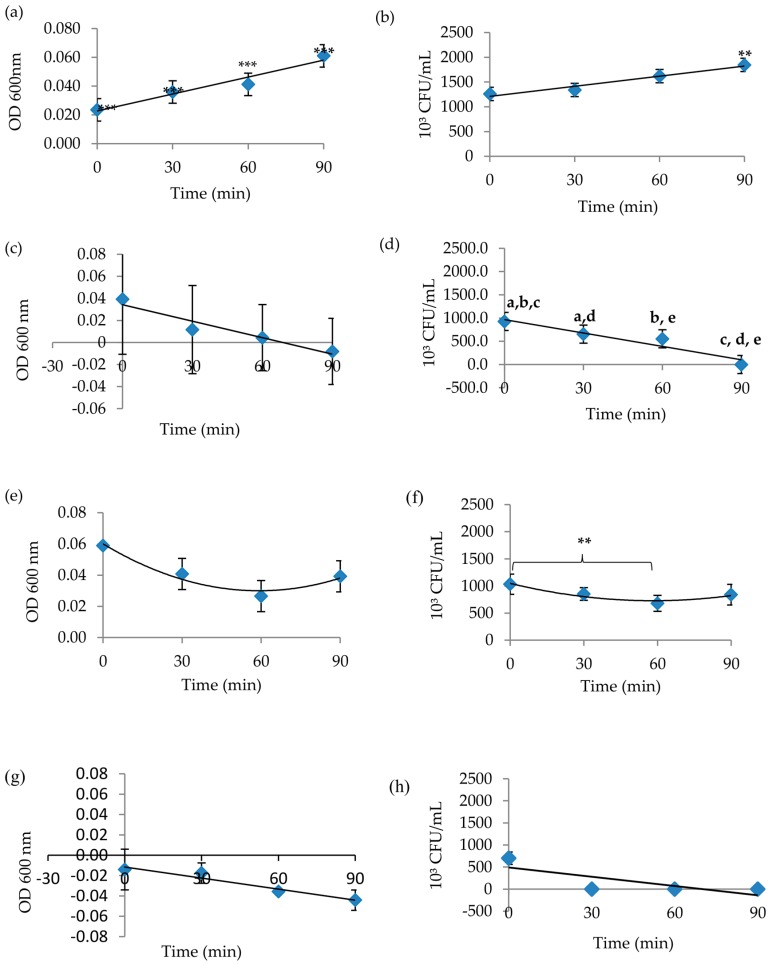
*P. aeruginosa* growth curve for the susceptibility test after exposure to 0.4 g/mL of extracts from 0 to 90 min. (**a**) control optical density (OD) values, (**b**) control viable count values, (**c**) cinnamon aqueous extract OD values, (**d**) cinnamon aqueous extract viable count values, (**e**) kaffir lime aqueous extract OD values, (**f**) kaffir lime aqueous extract viable count values, (**g**) lime juice OD values, and (**h**) lime juice viable count values. The post hoc Tukey HSD test showed that the control OD values were very significantly different from each other at all time points (*** *p* < 0.00), whereas the control viable count value at 90 min was significantly different from values at all other time points (** *p* < 0.01). The viable cell counts of *P. aeruginosa* exposed to the cinnamon aqueous extract differed significantly from each other at all time point (dots with same letter differ significantly, ** *p* < 0.01), except for time points between 30 min and 60 min. For the kaffir lime aqueous extract, viable count values at 0 min differed significantly from those at 60 min (** *p* < 0.01). Results were expressed in OD (600 nm) and 10³ CFU/mL, respectively, with *n* = 3.

The bacteriostatic effects of key lime juice and aqueous extracts of cinnamon bark and kaffir lime leaves on 10^3^ cells/mL *Pseudomonas aeruginosa* and methicillin resistance *Staphylococcus aureus* (*MRSA*) were determined using a spectrophotometer (for optical density (OD) values) and the standard method of viable counts on agar as a reference. The *P. aeruginosa* positive control exhibited a smooth increase in the graph pattern for both OD and viable counts, which shows kinetic growth of healthy bacteria cells (Figure 1). Microorganisms were incubated with agitation in order to mix and enhance the growth of the bacteria. This helped to prevent cells from forming biofilms and aggregates. Bacterial growth increases with time until it reaches a plateau, indicating the cessation of bacterial growth due to nutrient depletion.

OD values for the cinnamon aqueous extract showed a sharp decrease over time, which was also apparent in the viable count data (10^3^ CFU/mL) (Figure 1c,d). *P. aeruginosa* was susceptible to cinnamon at 90 min, at which time a negative OD value was detected and no viable cells were counted. The decrease in OD represented the death phase of *P. aeruginosa*. Both methods reflected a correlation in sustaining the sensitivity range of the *P. aeruginosa* with a constant concentration of the extracts sufficient for inhibiting bacteria in standardised turbidity of cell suspension. The kaffir lime aqueous extract treatment showed a smooth decline in absorbance over time, with a slight increase at 90 min. This pattern reflected the viable count result (Figure 1e,f). Inhibition of *P. aeruginosa* occurred between 0 to 60 min, which means that the kaffir lime aqueous extract had a bacteriostatic effect in this time range, but the effect weakened by 90 min. Lime juice had excellent bactericidal activity against *P. aeruginosa*, as indicated by the constant decrease in absorbance and the elimination of viable counts at the end of the experiment (Figure 1g,h).

**Figure 2:**
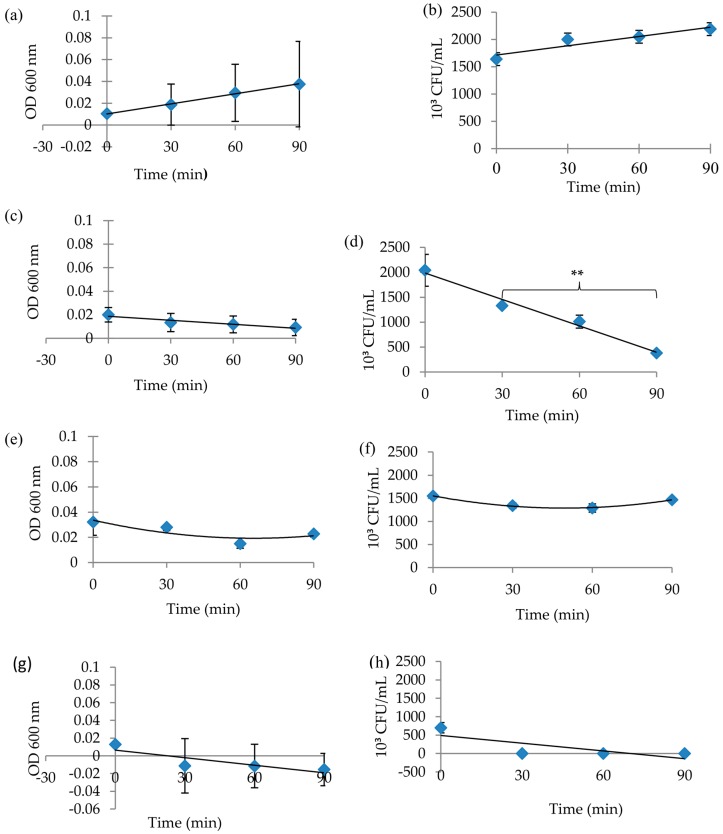
Growth curve of methicillin resistant *Staphylococcus aureus* (*MRSA*) for the susceptibility test after exposure to 0.4 g/mL of extract from 0 to 90 min. (**a**) control OD values, (**b**) control viable count values, (**c**) cinnamon aqueous extract OD values, (**d**) cinnamon aqueous extract viable count values, (**e**) kaffir lime aqueous extract OD values, (**f**) kaffir lime aqueous extract viable count values, (**g**) lime juice OD values, and (**h**) lime juice viable count values. The Dunnett T3 test indicated that the viable count values for the cinnamon aqueous extract at 30 min and 90 min differed significantly from each other (** *p* < 0.01). Results were expressed in OD (600 nm) and 10³ CFU/mL, respectively, with *n* = 2.

The *MRSA* OD values of the positive control increased linearly, and the viable count values showed a slow increase (Figure 2a,b). OD and the viable cell counts of *MRSA* cells exposed to the cinnamon aqueous extract exhibited a constant decline (Figure 2c,d). OD and viable cell counts of *MRSA* cells that were exposed to the kaffir lime aqueous extract decreased at first and then increased at 90 min (Figure 2e,f). Thus, this extract had a bacteriostatic effect for the first 60 min, but the effect weakened thereafter. *MRSA* cells that were exposed to lime juice showed a sharp decline in OD values and viable cell counts over the duration of the experiment (Figure 2g,h).

### 2.2. Preliminary Studies of Extracts as Potential Cleaning Agents

Table 1 shows that the total clearance percentage of bacteria for lime juice was 86.7% for 15 sampled surfaces using the TSA contact agar plate, and this value was marginally higher than that of detergent (80%). For detergent, 1 of 15 (6.7%) sampled surfaces showed reduction of bacteria. The proportion of sample surfaces with no bacterial colonies for both detergent and lime juice was 13.3%. Lime juice produced a better clearance rate than the commercial disinfectant detergent, which shows that *C. aurantifolia* juice is a competent cleaning agent.

The Gram stain analysis results (Table 2) demonstrated that the sanitation of the laboratories before intervention was in the marginal category, as Gram negative rod bacteria were not present in those laboratories. However, there were several gram positive cocci and rod bacteria that were found inhabiting in the environment of the laboratories. Most samples reached excellent sanitation after intervention, and only one fall under marginal sanitation due to the presence of gram positive rod identified to be *Bacillus* spp., a facultative anaerobe bacterium.

## 3. Discussion

In the control treatment, both *P. aeruginosa* and *MRSA* exhibited healthy growth, indicating that the environmental requirements for growth had been met in the experiment. Of the extracts that were tested, *P. aeruginosa* and *MRSA* were most sensitive to lime juice, followed by the cinnamon aqueous extract and the kaffir lime aqueous extract. Lime juice exhibited excellent bactericidal activity against *P. aeruginosa* throughout the duration of the experiment, and *MRSA* was susceptible to lime juice after 30 min (i.e., no growth in the viable count analysis). The cinnamon aqueous extract had a bacteriostatic effect for the first 60 min of the experiment and successfully eliminated *P. aeruginosa* for the subsequent time based on results of the count analysis. *MRSA* was sensitive to the cinnamon extract, as decreasing growth and variable count values were observed. Cinnamon exhibited better antimicrobial activity against *P. aeruginosa* when compared to *MRSA*. *P. aeruginosa* and *MRSA* were susceptible to the kaffir lime extract for the first 60 min of the experiment, but the bacteriostatic effect weakened by 90 min, as shown by elevated growth rates. Both types of bacteria might have developed a special metabolic response to overcome and resist the kaffir lime extract after exposure to it for a period of time [5].

The spectrophotometric method is essential for obtaining total cell mass data from turbidity measurements. Cell mass is proportional to the number of cells. Sensitivity of spectrophotometry analysis is optimal at shorter wavelength, whereas longer wavelength is best for cell density measurement [6]. Moreover, because each bacterium has a different mass, the OD reading varies among different bacteria species. Future studies should focus on developing a technique to identify the OD that is most suitable for each bacteria type. Herein, *P. aeruginosa* had a higher OD reading when compared to *MRSA*, possibly because of the former’s better proliferation rate. *P. aeruginosa* proliferates better in humid conditions, whereas *MRSA* multiplies better at lower humidity [7]. A negative OD value indicates that bacterial cytoplasmic membranes have collapsed and lysed due to the addition of bactericidal agents, such as citrus and cinnamon [6]. In the current study, the OD dropped dramatically when cells were exposed to lime juice.

TSA contact agar plates are used for the isolation and cultivation of microorganisms [8]. However, some types of fastidious microorganisms, viruses, and fungi are not detectable using this method. A specific medium and factors (e.g., temperature) are needed to enhance growth of such species [6,8]. Therefore, the absence of microbial colonies before intervention is not necessarily a true negative result. The presence of Gram negative rod bacteria is indicative of poor sanitation and a very unhealthy environment in which to live and work. In this study, most of the samples from the three laboratories tested fell into the marginal sanitation category prior to intervention. This finding indicates that those laboratories were free of Gram negative bacteria, which are the main pathogenic agents that are responsible for causing disease, such as bacteraemia and NI associated infection [6].

After the disinfection treatment, one sample was in the marginal sanitation category. Gram positive rod bacteria were found on this surface in countable numbers after the disinfectant treatment, perhaps because this was a heavily used surface that likely contained organic matter, such as carbon, that contributed to the growth of bacteria [6]. Additionally, different microorganisms have different adhesion properties, which can switch between being reversible and irreversible. Bacterial strains that do not have slimy properties and have reversible adhesion are less likely to be pathogenic. Bacteria are less able to adhere to smoother surfaces, such as glass, as compared to plastic surfaces. Irreversible adhesion occurs during the first stage of biofilm (slime) formation. Bacteria can replicate inside biofilms, causing biofilm mass to increase. Biofilm formation depends on parameters, such as humidity and temperature [9]. The presence of biofilms makes the cleaning process difficult. If the surface is unhygienic and organic material is present (i.e., fuel for microorganisms), bacteria will accumulate on the surface and proliferate. Thus, before using disinfectant, it is advisable to wash and clean the surface to remove dirt [9].

The disinfectant detergent that was used in this experiment is capable of both cleaning and disinfecting. However, its use has side effects, such as allergies, eczema, and asthma [3,4]. Lime juice was previously shown to have antimicrobial activities due to the presence of bioactive compounds, such as phenolics, which can inhibit the growth of most food-borne and food spoilage microorganisms [10] via compounds that induce enzyme detoxification and regulate the immune response [11]. The results reported herein show that lime juice has bactericidal activities and can productively remove microorganisms from fomite surfaces. Further studies should focus on identifying the bioactive compounds present in lime juice and their potential for use in chemical and pharmacological applications [12].

## 4. Materials and Methods

### 4.1. Plant Materials

*C. iners* bark, *C. aurantifolia* fruits, and *C. hystrix* leaves were collected from Bertam, Kepala Batas, Malaysia. The plants were authenticated by a taxonomist, and voucher specimens were deposited in the Integrative Medicine Cluster Herbarium Collection.

### 4.2. Aqueous and Juice Extraction

For bark and leaf samples, one part dried plant (20 g) in five parts of sterilized water (50 mL) was boiled and reduced to one-fifth of the original volume. The mixture was autoclaved at 100 °C for 15 min for sterilisation [13]. *C. aurantifolia* fruits were wiped clean with 70% ethanol and distilled water. The fruits were cut in half with a sterilised knife and aseptically squeezed into a sterile beaker. The extracts and juice were centrifuged at 3500× g for 20 min to remove undesired residue. Supernatant was collected and stored at −20 °C until used in the experiments [12,14,15].

### 4.3. Bacteria Susceptibility Test

The two strains of bacteria used in this study were obtained from the American Type Culture Collection (ATCC) [16]. *P. aeruginosa* (ATCC 27853) and methicillin resistant *Staphylococcus aureus (MRSA*, ATCC 33591) were kept in the microbiology laboratory of the Clinical Trial Centre, Advanced Medical and Dental Institute (AMDI), Universiti Sains Malaysia (USM). Colonies were inoculated into the nutrient broth using a sterilised loop. The broth was mixed thoroughly using a vortex to prevent aggregation, followed by incubation overnight at 37 °C in an incubator shaker [17]. Each cultured bacteria suspension was diluted via 10-fold serial dilution. For each species, a dilution of 10^3^ cells/mL was prepared for the control and test samples. Cinnamon and kaffir lime aqueous extracts at 0.4 g/mL and lime juice were evaluated in the susceptibility test using a quantity of 2 mL [18]. Control and test cultures were incubated with agitation at 37 °C. Absorbance was measured using a UV-VIS spectrophotometer (Perkin Elmer, Lambda 25, Norwalk, CT, USA), and a 50 µL aliquot of each suspension was aseptically plated onto an agar plate to obtain viable counts. OD readings and plating analysis were conducted at 0, 30, 60, and 90 min [17,19].

### 4.4. Fomite Surface Sampling and Gram Staining

Five surfaces of inanimate objects were randomly selected from the laboratories and marked [20]. The test surface was moistened with distilled water to facilitate the dispersion of bacterial clumps and result in a higher count. Moreover, a dry surface could it make difficult to transfer bacteria to the agar due to surface charge, topography, and hydrophobicity [21]. The control treatment contained only distilled water and was sprayed on the test surface and left for 5 min before sampling. A similar procedure was utilised for the test treatment, which consisted of disinfectant detergent and lime juice. After spraying the surface with the control or test treatment and allowing it to sit for 5 min, the test surface was wiped and cleaned with distilled water. An agar plate was applied to the surface for 5 s and then incubated at 35 °C for 24 h [21,22]. The number of colony forming units (CFU) per area sampled = CFU/cm^2^ was calculated to determine % effectiveness. The % effectiveness before (control) and after disinfection was calculated using the following formula [23]:

$$\frac{{\mathrm{Before}/\mathrm{after}\;\mathrm{test}\;(\mathrm{CFU}/\mathrm{cm}}^{2})}{{\mathrm{Total}\;\mathrm{before}/\mathrm{after}\;\mathrm{test}\;(\mathrm{CFU}/\mathrm{cm}}^{2})}\times 100\%$$

To examine the contents of the agar plates, a colony from a given sample was smeared onto a glass slide and dried near a flame. The slide was flooded with crystal violet solution for 1 min and rinsed with tap water for 2 s. The slide then was flooded with Gram’s Iodine solution (mordant) and alcohol to decolourise the sample. Safranin solution (counterstain) was added and rinsed with water after 30 s. The slide was allowed to dry on a slide warmer and then examined under an oil-immersed microscope (1000×) [24].

### 4.5. Statistical Analysis

Data were analysed using Statistical Package for the Social Sciences (IBM SPSS version 22.0, Armonk, NY, USA). One way ANOVA Dunnett T3 test and post hoc Tukey HSD test were used to determine the significance of samples at different time point where ** *p* < 0.01; *** *p* < 0.00.

## 5. Conclusions

*C. aurantifolia* juice is a potential bactericidal agent that exhibits strong and effective bactericidal activity against *P. aeruginosa* and *MRSA* and is not affected by organic material. Thus, lime juice could be used as an effective disinfectant. Future studies of its use in cleaning intervention should include sampling from clinics and hospitals and different types of surfaces. Domestic household and hospital employees could benefit from development of a cleaning product containing *C. aurantifolia* juice.

## Figures and Tables

**Table 1:** Effectiveness of commercial disinfectant detergent and *C. aurantifolia* juice (%) as cleaning agents.

	Null (%)	Reduction (%)	Clearance (%)	Total
Disinfectant detergent (*n* = 15)	13.3	6.7	80.0	100
*C. aurantifolia* juice (*n* = 15)	13.3	–	86.7	100

**Table 2:** Gram stain test to determine the presence of Gram positive bacteria and Gram negative bacteria on the experimental surface.

Excellent Sanitation		Marginal Sanitation		Unsatisfactory Sanitation	
Before	After	Before	After	Before	After
*n* = 30	*n* = 30	*n* = 30	*n* = 30	*n* = 30	*n* = 30
4	29	26	1	0	0
